# Suboptimal ceftazidime-avibactam exposure drives sequential *bla*_KPC_ mutations and intra-host coexistence of *Klebsiella pneumoniae* harboring distinct variants leading to persistent infection

**DOI:** 10.1128/spectrum.03308-25

**Published:** 2026-02-20

**Authors:** Feng Yang, Weiwei Yang, Jinghao Zhang, Chengkang Tang, Li Ding, Siquan Shen, Yanhao Zhou, Hongyu Zhao, Jiaojiao Zhou, Fupin Hu

**Affiliations:** 1 Institute of Antibiotics, Huashan Hospital, Fudan University 198171, Shanghai, China; 2 Department of Laboratory Medicine, Huadong Hospital, Fudan University 12478, Shanghai, China; 3 Joint Laboratory of Hospital & Enterprise for Pathogen Diagnosis of Drug-resistant Bacterial Infections and Innovative Drug R&D, Shanghai, China; 4 Key Laboratory of Clinical Pharmacology of Antibiotics, Ministry of Health, Shanghai, China; 5 Department of Respiratory and Critical Care Medicine, Huadong Hospital, Fudan University 12478, Shanghai, China; Universita degli Studi dell'Insubria, Varese, Italy

**Keywords:** *Klebsiella pneumoniae*, KPC variants, *bla*_KPC _evolution, intra-host coexistence, ceftazidime-avibactam, reduced-dose therapy

## Abstract

KPC-producing *Klebsiella pneumoniae* infections are a rising global health threat. The factors driving *bla*_KPC_ mutations during treatment and their contribution to infection persistence remain unclear. This study characterized *bla*_KPC_ evolution under reduced-dose ceftazidime-avibactam therapy for renal impairment, using 10 sequential *K. pneumoniae* isolates (showing resistance shifts) collected from a single patient during a persistent infection. Antimicrobial susceptibility was determined by broth microdilution. Whole-genome sequencing was used to investigate *bla*_KPC_ evolution. Time-kill assays assessed bactericidal and selective effects. Plasmid transformation and cloning assays validated *bla*_KPC_ phenotypes. Four *bla*_KPC_ subtypes (*bla*_KPC-2_, *bla*_KPC-33_, *bla*_KPC-71_, and *bla*_KPC-145_) were identified. Functional validation confirmed that *bla*_KPC-2_ conferred carbapenem resistance, while the three variants mediated ceftazidime-avibactam resistance. Tracing their evolutionary trajectory revealed seven sequential mutation events during dose-adjusted ceftazidime-avibactam and carbapenem therapy, ultimately yielding the four observed subtypes. These mutations appeared to accumulate stepwise from *bla*_KPC-2_ to *bla*_KPC-33_ (D179Y) and *bla*_KPC-145_ (D179Y, T264A), which progressively increased ceftazidime-avibactam MICs. Molecular docking predicted variant-specific alterations in avibactam binding. Time-kill assays showed that 1×MIC/sub-MIC ceftazidime–avibactam suppressed but failed to eradicate the *bla*_KPC-2_-harboring isolate, potentially facilitating *bla*_KPC-2_ evolution. Multiple *bla*_KPC_ variants coexisted within the same specimen, indicating intra-host heterogeneity. These reversible *bla*_KPC_ mutations disappeared after 8 days of combined imipenem and ceftazidime-avibactam therapy. Meropenem-vaborbactam, imipenem-relebactam, and aztreonam-avibactam showed *in vitro* activity against both KPC- and KPC variant-producing isolates. In conclusion, suboptimal ceftazidime-avibactam exposure may promote sequential *bla*_KPC-2_ mutations and intra-host variants coexistence, driving persistent infection. These findings highlight the urgent need for improved therapeutic strategies against KPC/KPC variant-producing strains.

**IMPORTANCE:**

*Klebsiella pneumoniae*, especially those producing KPC or KPC variant enzymes, represent a growing threat to global health due to limited treatment options and frequent therapeutic failure. The emergence of *bla*_KPC_ mutations during treatment further complicates therapy, as these mutations can lead to shifts in resistance profiles. Understanding the patterns and driving factors behind *bla*_KPC_ mutations is crucial for developing more effective treatment strategies. This study shows that dose reduction of ceftazidime-avibactam during treatment for renal impairment can promote potential stepwise mutations in *bla*_KPC_, leading to the emergence of multiple variants with corresponding resistance shifts. Moreover, insufficient ceftazidime-avibactam and carbapenems fail to eradicate the bacteria, allowing the coexistence of *K. pneumoniae* subpopulations carrying distinct *bla*_KPC_ variants within a single patient. The findings highlight the need for individualized treatment regimens and optimized dosing and combination strategies to limit the emergence and persistence of KPC variant-producing *K. pneumoniae* during therapy.

## INTRODUCTION

Infections caused by carbapenem-resistant *Klebsiella pneumoniae* (CRKP) represent an escalating public health threat globally and are associated with high mortality rates (1). In CRKP, carbapenem resistance is primarily mediated by carbapenemases, which inactivate carbapenems through hydrolysis of the β-lactam ring (2). *Klebsiella pneumoniae* carbapenemase (KPC) is one of the most prevalent carbapenemases, with KPC-2 and its variants predominantly reported in East Asia and the USA, whereas KPC-3 and its variants are more frequently detected in the Americas and Europe (3, 4). β-Lactam/β-lactamase inhibitor combinations are currently preferred treatment options for infections caused by KPC-producing CRKP (5). Since its approval in 2015, ceftazidime-avibactam has become a principal therapy for CRKP infections, demonstrating favorable clinical efficacy. However, despite the effectiveness, ceftazidime-avibactam exerts selective pressure on *bla*_KPC_, leading to the emergence of diverse KPC variants (6, 7).

To date, more than 260 *bla*_KPC_ variants have been documented in the NCBI database, with a marked increase following the clinical introduction of ceftazidime-avibactam. While ceftazidime-avibactam exposure has been recognized as the predominant driver of *bla*_KPC_ mutations (3, 6), growing evidence suggests that pneumonia, respiratory or rectal colonization, and immunocompromised states related to lung or kidney transplantation may further facilitate the emergence and selection of *bla*_KPC_ variants (8–10). Many of these variants harbor mutations in structural hot spots, including the Ω-loop (residues 164–179), loop 237–243, and loop 267–275, which are critical for antibiotic binding. Such mutations often confer ceftazidime-avibactam resistance while restoring susceptibility to certain carbapenems (3). However, treatment with these seemingly susceptible carbapenems may lead to reversion of KPC variants to wild-type forms, regaining carbapenem resistance (7, 11, 12). These dynamic shifts in resistance phenotypes among KPC- and KPC-variant-producing CRKPs pose significant challenges for antimicrobial therapy. While intra-host *bla*_KPC_ mutations arising during treatment have been frequently reported (11, 13), consecutive mutations within the same patient are uncommon, and their clinical and molecular characteristics under antibiotic pressure remain poorly understood. In this study, we investigated a case of persistent CRKP infection, providing insights into the intra-host dynamics of *bla*_KPC_ mutations and coexistence, as well as their implications for antimicrobial treatment.

## RESULTS

### Clinical isolates and antimicrobial susceptibility profiles

**Fig 1:**
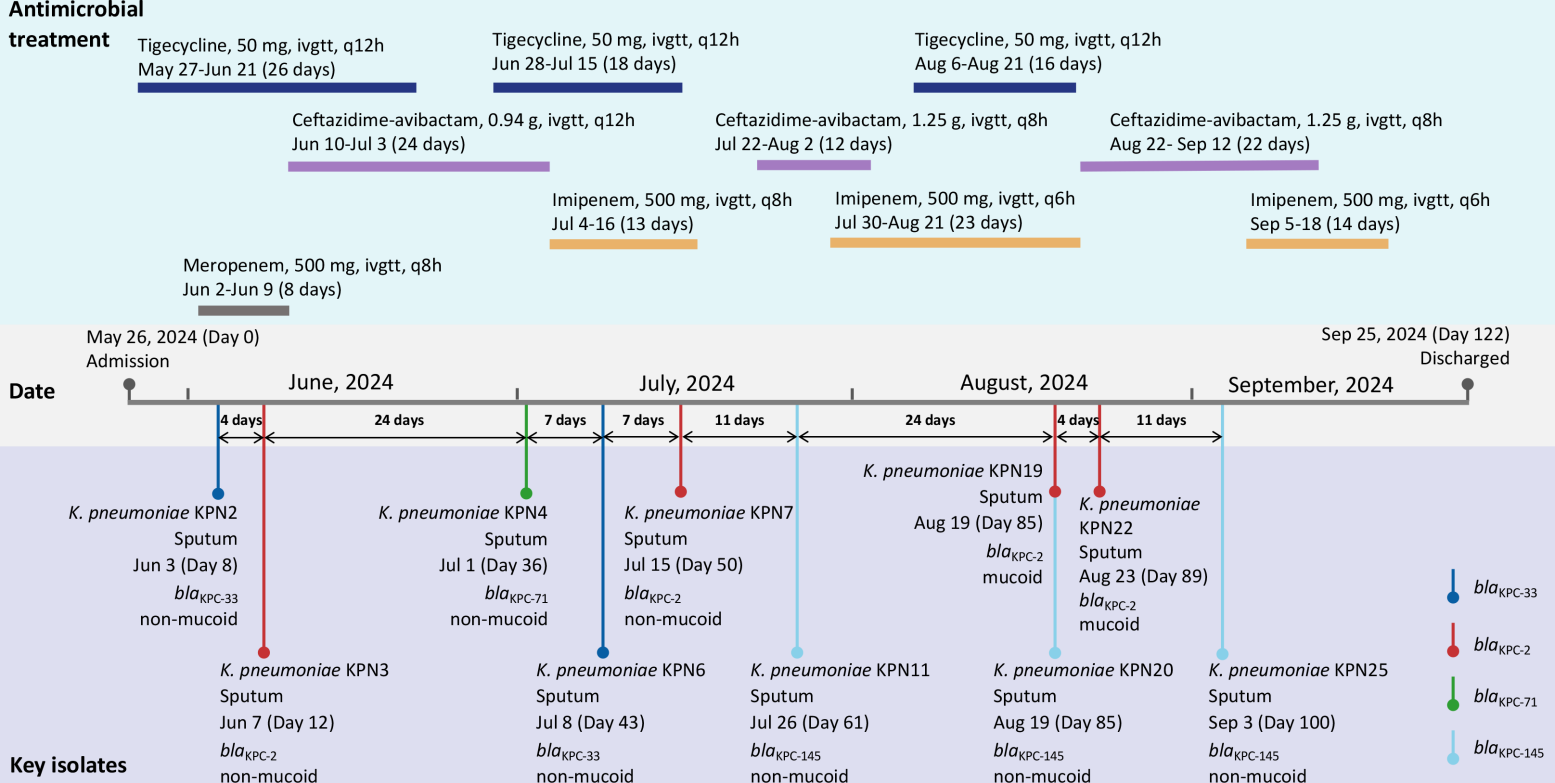
Clinical timeline of antimicrobial therapy and key *K. pneumoniae* isolates during hospitalization.

During the 122-day hospitalization, *K. pneumoniae* was repeatedly isolated from the sputum specimens, yielding 10 non-duplicate isolates from the single patient (Fig. 1; Table 1). Each pair of sequential isolates exhibited changes in antimicrobial resistance profiles. The first isolate (*K. pneumoniae* KPN2) was carbapenem-susceptible, with an imipenem MIC of 1 mg/L and a ceftazidime-avibactam MIC of >64 mg/L. Subsequently, sputum cultures alternately yielded imipenem-resistant/ceftazidime-avibactam-susceptible isolates (*K. pneumoniae* KPN3, *K. pneumoniae* KPN7, *K. pneumoniae* KPN19, and *K. pneumoniae* KPN22), with imipenem MICs of 64 mg/L and ceftazidime-avibactam MICs of 1–4 mg/L, and imipenem-susceptible/ceftazidime-avibactam-resistant isolates (*K. pneumoniae* KPN4, *K. pneumoniae* KPN6, *K. pneumoniae* KPN11, *K. pneumoniae* KPN20, and *K. pneumoniae* KPN25), with imipenem MICs of 0.25–1 mg/L and ceftazidime-avibactam MICs ≥64 mg/L. Antimicrobial therapy was adjusted according to susceptibility profiles, and ceftazidime-avibactam and carbapenem regimens were administered at reduced doses because of renal impairment (Fig. 1). Notably, after an 8-day imipenem and ceftazidime-avibactam combination therapy near the end of hospitalization, no further resistance shifts were observed. The patient showed gradual clinical improvement and was discharged on hospital day 122, with sputum cultures turning negative prior to discharge. During the same hospitalization period, no *K. pneumoniae* or other bacterial isolates exhibiting imipenem susceptibility and ceftazidime–avibactam resistance similar to that identified in this case were recovered from other patients in the ward or adjacent units. This suggests that these resistance dynamics arose within this individual patient rather than being transmitted throughout the ward.

**TABLE 1:** MICs of antimicrobial agents for the 10 clinical *K. pneumoniae* strains^*a*^

Antimicrobial agents	MIC (mg/L)									
	KPN2 (*bla*_KPC-33_)	KPN3 (*bla*_KPC-2_)	KPN4 (*bla*_KPC-71_)	KPN6 (*bla*_KPC-33_)	KPN7 (*bla*_KPC-2_)	KPN11 (*bla*_KPC-145_)	KPN19 (*bla*_KPC-2_)	KPN20 (*bla*_KPC-145_)	KPN22 (*bla*_KPC-2_)	KPN25 (*bla*_KPC-145_)
Imipenem	1 (S)	64 (R)	0.25 (S)	0.5 (S)	64 (R)	1 (S)	64 (R)	0.25 (S)	64 (R)	0.25 (S)
Imipenem-relebactam	0.125 (S)	0.5 (S)	0.125 (S)	0.125 (S)	1 (S)	0.25 (S)	1 (S)	0.125 (S)	0.5 (S)	0.25 (S)
Meropenem	2 (I)	>64 (R)	1 (S)	2 (I)	>64 (R)	2 (I)	>64 (R)	1 (S)	>64 (R)	2 (I)
Meropenem-vaborbactam	1 (S)	1 (S)	0.5 (S)	1 (S)	1 (S)	2 (S)	4 (S)	1 (S)	4 (S)	2 (S)
Ceftazidime	>64 (R)	>64 (R)	>64 (R)	>64 (R)	>64 (R)	>64 (R)	>64 (R)	>64 (R)	>64 (R)	>64 (R)
Ceftazidime-avibactam	>64 (R)	1 (S)	>64 (R)	64 (R)	2 (S)	>64 (R)	2 (S)	>64 (R)	4 (S)	>64 (R)
Cefepime	>32 (R)	>32 (R)	>32 (R)	>32 (R)	>32 (R)	>32 (R)	>32 (R)	>32 (R)	>32 (R)	>32 (R)
Aztreonam	>64 (R)	>64 (R)	>64 (R)	>64 (R)	>64 (R)	>64 (R)	>64 (R)	>64 (R)	>64 (R)	>64 (R)
Aztreonam-avibactam	2 (S)	1 (S)	2 (S)	2 (S)	1 (S)	2 (S)	1 (S)	2 (S)	2 (S)	2 (S)
Amikacin	>64 (R)	>64 (R)	>64 (R)	>64 (R)	>64 (R)	>64 (R)	>64 (R)	>64 (R)	>64 (R)	>64 (R)
Ciprofloxacin	>8 (R)	>8 (R)	>8 (R)	>8 (R)	>8 (R)	>8 (R)	>8 (R)	>8 (R)	>8 (R)	>8 (R)
Eravacycline	0.5 (S)	0.5 (S)	0.5 (S)	0.5 (S)	1 (S)	0.5 (S)	1 (S)	1 (S)	1 (S)	1 (S)
Tigecycline	1 (S)	0.5 (S)	0.5 (S)	0.5 (S)	1 (S)	1 (S)	1 (S)	1 (S)	1 (S)	1 (S)
Colistin	0.5 (S)	0.5 (S)	1 (S)	0.5 (S)	1 (S)	0.5 (S)	0.5 (S)	0.5 (S)	0.5 (S)	0.5 (S)
Trimethoprim- sulfamethoxazole	>16 (R)	>16 (R)	>16 (R)	>16 (R)	>16 (R)	>16 (R)	>16 (R)	>16 (R)	>16 (R)	>16 (R)

^*a*^ For agents without CLSI criteria, aztreonam-avibactam was interpreted using the EUCAST breakpoint, eravacycline using the ChiCAST breakpoint, and tigecycline using the FDA breakpoint.

Unlike imipenem, the 10 isolates showed inconsistent response against meropenem, with several strains susceptible to imipenem but showing intermediate resistance to meropenem (Table 1). Beyond carbapenems and ceftazidime-avibactam, all isolates showed consistent resistance to ceftazidime (MICs >64 mg/L), cefepime (MICs >32 mg/L), aztreonam (MICs >64 mg/L), amikacin (MICs >64 mg/L), ciprofloxacin (MICs >8 mg/L), and trimethoprim-sulfamethoxazole (MICs >16 mg/L). They remained susceptible to meropenem-vaborbactam (MICs 0.5–4 mg/L), imipenem-relebactam (MICs 0.125–1 mg/L), aztreonam-avibactam (MICs 1–2 mg/L), eravacycline (MICs 0.5–1 mg/L), tigecycline (MICs 0.5–1 mg/L), and colistin (MICs 0.5–1 mg/L). In addition, efflux pump inhibition assays showed that MICs of imipenem, meropenem, or ceftazidime-avibactam in resistant isolates were largely unchanged in the presence of the efflux pump inhibitor, suggesting that efflux pumps did not play a detectable role in the observed resistance phenotypes (Table S1). Of the 10 isolates, 8 exhibited a non-mucoid colony morphology. Meanwhile, *K. pneumoniae* KPN19 and *K. pneumoniae* KPN22 (both carrying *bla*_KPC-2_) showed a mucoid phenotype and a positive string test result (Fig. 1; Fig. S1 for representative images). Despite two isolates exhibiting a mucoid phenotype, no genotypic differences associated with the hypervirulence or hypermucoviscosity determinants reported were identified. All 10 isolates shared nearly identical virulence and capsule gene profiles.

### Genetic profiles of 10 *K. pneumoniae* isolates

**Fig 2:**
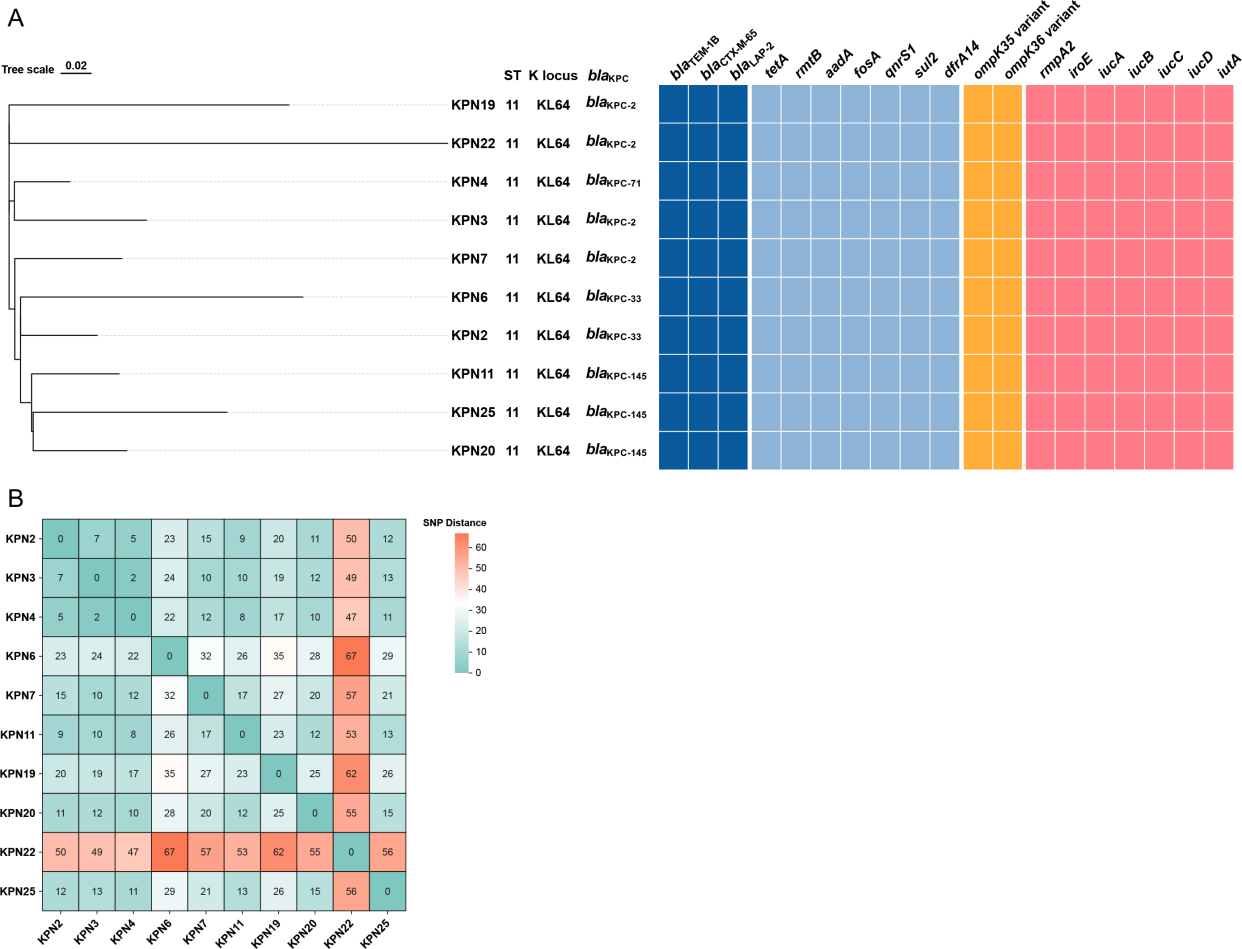
Genomic and phylogenetic profiles of 10 clinical *K. pneumoniae* isolates. (**A**) Maximum-likelihood phylogeny of 10 clinical *K. pneumoniae* isolates, annotated with sequence types (STs), capsular (K) loci, and the presence of key antimicrobial resistance and virulence genes. The *ompK35* variant harbors p.Lys28fs, and the *ompK36* variant harbors p.134_135insGlyAsp. (**B**) Pairwise SNP distances between isolates.

Among the 10 *K. pneumoniae* isolates, different *bla*_KPC_ alleles were identified, consistent with their imipenem and ceftazidime-avibactam susceptibility profiles. *bla*_KPC-2_ was present in *K. pneumoniae* KPN3, *K. pneumoniae* KPN7, *K. pneumoniae* KPN19, and *K. pneumoniae* KPN22 (imipenem-resistant/ceftazidime-avibactam-susceptible), while *bla*_KPC-33_ (*K. pneumoniae* KPN2, *K. pneumoniae* KPN6), *bla*_KPC-71_ (*K. pneumoniae* KPN4), and *bla*_KPC-145_ (*K. pneumoniae* KPN11, *K. pneumoniae* KPN20, *K. pneumoniae* KPN25) were detected in the imipenem-susceptible/ceftazidime-avibactam-resistant group (Fig. 2A; Table 1). Further functional validation assays supported these genotype–phenotype associations. In plasmid transformation assays, the *bla*_KPC-2_ transconjugant showed ≥16-fold increases in imipenem and meropenem MICs, while transconjugants carrying *bla*_KPC-33_, *bla*_KPC-71_, or *bla*_KPC-145_ showed >64-fold increases in ceftazidime-avibactam MICs (Table 2). Similarly, the *bla*_KPC-2_ clone led to >16-fold increases in imipenem and meropenem MICs, while *bla*_KPC-33_, *bla*_KPC-71_, or *bla*_KPC-145_ clones conferred ≥64-fold increases in ceftazidime-avibactam MICs. Together, these findings suggested that *bla*_KPC-2_ was the key determinant of carbapenem resistance, and *bla*_KPC-33_, *bla*_KPC-71_, and *bla*_KPC-145_ were key determinants of ceftazidime-avibactam resistance in *K. pneumoniae*.

**TABLE 2:** MICs (mg/L) of antimicrobial agents for representative transconjugants and clone strains corresponding to the four KPC variants in this study^*a*^

Antimicrobial agents	*E. coli* DH5α-transconjugant					*E. coli* DH5α-clone				
	DH5α	KPN2-T (*bla*_KPC-33_)	KPN3-T (*bla*_KPC-2_)	KPN4-T (*bla*_KPC-71_)	KPN11-T (*bla*_KPC-145_)	pHSG398	pKPC-2	pKPC-33	pKPC-71	pKPC-145
Imipenem	0.125	0.25	2	0.125	0.125	≤0.06	8	0.125	≤0.06	≤0.06
Meropenem	≤0.03	0.06	1	0.06	0.06	≤0.03	2	≤0.03	≤0.03	≤0.03
Ceftazidime	≤0.06	>64	>64	>64	>64	0.25	16	>64	>64	>64
Ceftazidime-avibactam	≤0.03	2	0.06	4	16	0.125	0.25	16	8	32
Cefepime	0.5	8	4	4	4	≤0.06	8	0.25	2	0.5
Aztreonam-avibactam	≤0.03	0.06	0.125	0.06	0.06	≤0.03	0.125	≤0.03	≤0.03	0.125
Aztreonam	≤1	64	>64	>64	64	0.125	128	≤1	≤1	≤1
Amikacin	≤1	>64	>64	>64	>64	1	≤1	≤1	≤1	≤1
Ciprofloxacin	≤0.06	0.125	0.125	0.125	0.125	≤0.06	≤0.06	≤0.06	≤0.06	≤0.06
Tigecycline	≤0.06	0.125	0.125	0.125	0.125	0.125	0.125	0.125	0.125	0.125
Colistin	≤0.125	0.25	0.25	0.25	0.25	0.25	0.25	0.25	0.25	0.25
Trimethoprim-sulfamethoxazole	≤0.06	0.5	0.5	0.5	0.5	≤0.06	≤0.06	≤0.06	≤0.06	≤0.06

^*a*^ In addition to *bla*_KPC_, the conjugative plasmids also carried other resistance genes, including *bla*_TEM-1B_, *bla*_CTX-M-65_, *bla*_LAP-2_, and *rmtB*. These co-transferred genes may contribute to the observed resistance phenotypes, particularly for aztreonam and amikacin.

In addition to *bla*_KPC_ variants, all isolates harbored three other β-lactamase genes, including *bla*_TEM-1B_, *bla*_CTX-M-65_, and *bla*_LAP-2_ (Fig. 2A). All isolates also carried resistance determinants *rmtB* and *aadA* (aminoglycosides), *qnrS1* (quinolone), *fosA* (fosfomycin), *sul2* (sulfonamides), and *dfrA14* (trimethoprim). Porin gene alterations were consistently observed, with all isolates carrying a frameshift in *ompK35* (p.Lys28fs) and an insertion in *ompK36* (p.134_135insGlyAsp), both predicted to reduce outer membrane permeability and contribute to elevated β-lactam MICs. Furthermore, several virulence-associated genes frequently reported in *K. pneumoniae*, including *rmpA2*, *iroE*, *iucA*, *iucB*, *iucC*, *iucD*, and *iutA*, were also detected (Fig. 2A). According to the PlasmidFinder analysis, all 10 isolates carried the same set of plasmid replicon types. The dominant replicons belonged to the IncFII family, including IncFII(pHN7A8) and IncFII(pSDP9R), which were both detected with 100% identity and coverage. IncFII-family plasmids are well-recognized vectors for *bla*_KPC_ and other β-lactamase genes, which is consistent with the carbapenemase background of these isolates. In addition, an IncHI1B(pNDM-MAR)-type replicon was detected with 99.15% identity and 473/570 bp coverage, indicating the presence of an IncHI1B-family plasmid backbone. Furthermore, screening of conjugation-associated genes revealed that none of the isolates carried a complete conjugative transfer region. Only partial *tra* genes (*traI*, *traC*, and *traM*) were detected, whereas essential T4SS *tra*/*trb* components were absent. These findings suggest that the plasmids are non-conjugative, which is consistent with the failure of our conjugation assays.

All 10 *K. pneumoniae* isolates belonged to ST11 and shared the same capsular type KL64 (Fig. 2A). These 10 sequential isolates were obtained from the same patient over time. Specifically, isolate 2 (*K. pneumoniae* KPN3) was collected 4 days after isolate 1 (*K. pneumoniae* KPN2); isolate 3 (*K. pneumoniae* KPN4), 24 days after *K. pneumoniae* KPN3; isolate 4 (*K. pneumoniae* KPN6), 7 days after *K. pneumoniae* KPN4; isolate 5 (*K. pneumoniae* KPN7), 7 days after *K. pneumoniae* KPN6; and isolate 6 (*K. pneumoniae* KPN11), 11 days after *K. pneumoniae* KPN7. Isolates 7 and 8 (*K. pneumoniae* KPN19 and *K. pneumoniae* KPN20) were recovered on the same day, 24 days after *K. pneumoniae* KPN11; isolate 9 (*K. pneumoniae* KPN22) was recovered four days after isolates *K. pneumoniae* KPN19 and 20; and isolate 10 (*K. pneumoniae* KPN25) was recovered 11 days after isolate *K. pneumoniae* KPN22 (Fig. 1). Pairwise SNP distances ranged from 2 to 67. Nine isolates were closely related (≤35 SNPs), supporting ongoing intra-host evolution. In contrast, the relatively higher divergence of *K. pneumoniae* KPN22 (47–67 SNPs from the others) suggests the possibility of distinct evolutionary events within the host rather than a single linear trajectory (Fig. 2B). Core-genome SNP-based phylogenetic analysis showed that the isolates clustered with relatively short branch lengths, indicating overall genomic similarity. Notably, isolates carrying the same *bla*_KPC_ alleles tended to group together, highlighting the association between *bla*_KPC_ variants and clonal lineage.

### Dynamic evolution of *bla*_KPC_ during treatment

**Fig 3:**
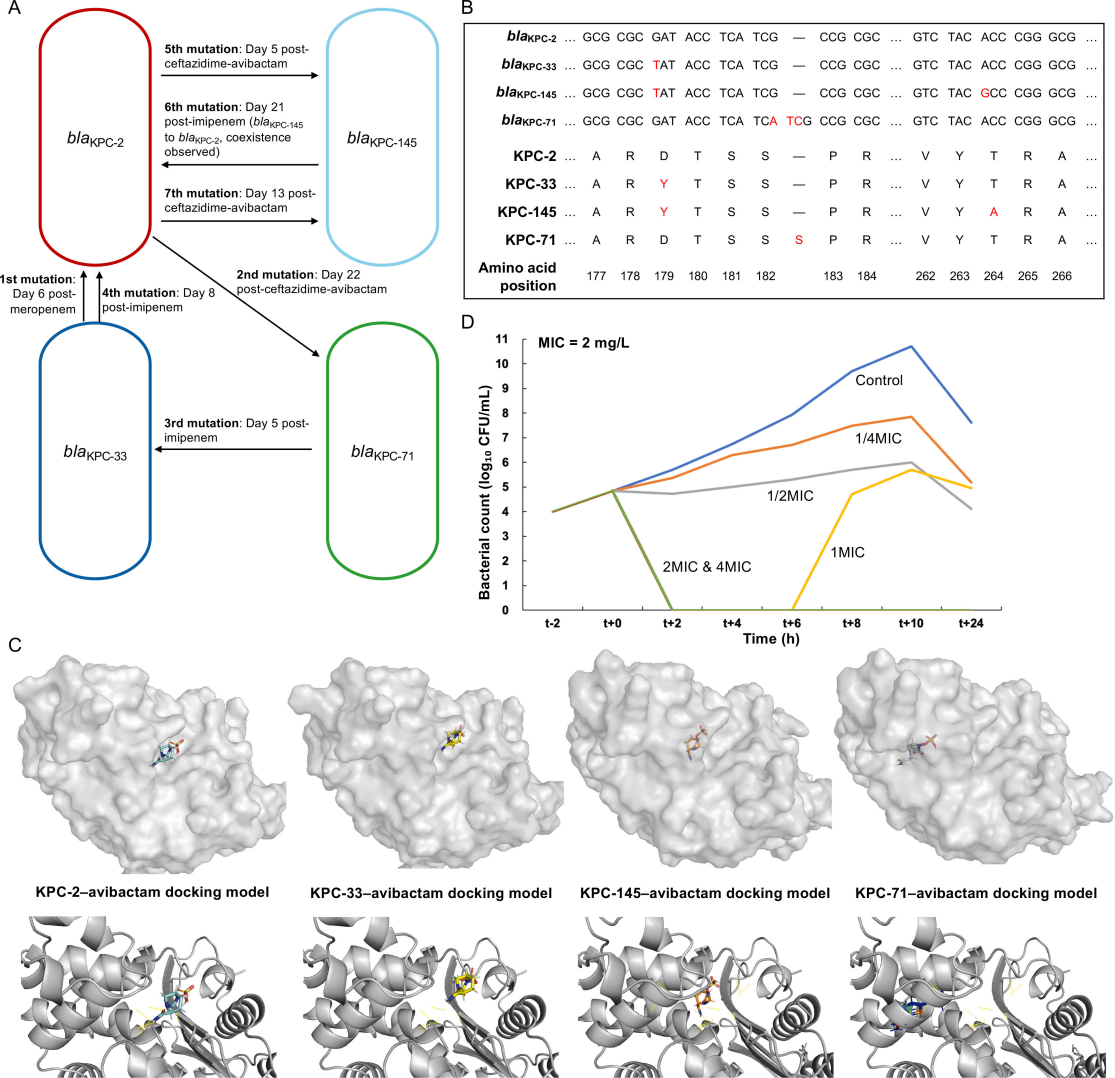
Sequential emergence of four *bla*_KPC_ variants in *K. pneumoniae* and the association with the application of antimicrobial agents. (**A**) Evolutionary pathway of *bla*_KPC_ variants in relation to the administration of specific antimicrobial agents. (**B**) The nucleotide and amino acid substitutions identified in the four *bla*_KPC_ variants in this study. (**C**) Molecular docking of avibactam with four KPC variants. For each KPC enzyme, two complementary visualizations of the predicted avibactam binding pose are shown. The upper panels display the surface view highlighting the position of avibactam within the binding pocket, while the lower panels present the cartoon representation with detailed ligand orientation and predicted interaction residues. For KPC-145 and KPC-71, partial transparency of the enzyme surface was applied to allow visualization of the ligand, as avibactam is otherwise deeply enclosed within the active-site. (**D**) Time-kill assay of ceftazidime-avibactam at various concentrations (4×MIC, 2×MIC, 1×MIC, 1/2×MIC, 1/4×MIC) against *bla*_KPC-2_-harboring *K. pneumoniae*.

Since all 10 isolates belonged to the same sequence type and showed close genetic relatedness, which evolved into four distinct *bla*_KPC_ subtypes during treatment, we further analyzed the temporal association between *bla*_KPC_ mutation and the administration of carbapenems or ceftazidime-avibactam. In chronological order, 7 mutation events were identified among the 10 isolates, including 1 instance of coexistence, where 2 isolates carrying different *bla*_KPC_ variants (KPN19 with *bla*_KPC-2_ and KPN20 with *bla*_KPC-145_) were obtained from the same specimen (Fig. 1 and 3A). Moreover, mutations from *bla*_KPC-2_ to variant alleles consistently occurred after ceftazidime-avibactam exposure (5–22 days later), while reversions from variant alleles back to *bla*_KPC-2_ occurred following meropenem or imipenem exposure (6–21 days later) (Fig. 3A). To better characterize the molecular basis of these evolutionary mutations, the nucleotide and amino acid sequences of the four *bla*_KPC_ subtypes were compared (Fig. 3B). The alignments revealed a possible stepwise accumulation of substitutions, in which *bla*_KPC-33_ differs from *bla*_KPC-2_ by a single nucleotide change (c.G537T, resulting in p.D179Y), while *bla*_KPC-145_ harbors an additional substitution based on *bla*_KPC-33_ (c.A792G, resulting in p.T264A). Plasmid transformation and cloning assays demonstrated that *bla*_KPC-145_ conferred a higher level of resistance to ceftazidime-avibactam than *bla*_KPC-33_. Specifically, the *bla*_KPC-145_ transconjugant showed a ceftazidime-avibactam MIC of 16 mg/L, compared with 2 mg/L for the *bla*_KPC-33_ transconjugant (Table 2). Similarly, the *bla*_KPC-145_ clone had a ceftazidime-avibactam MIC of 32 mg/L, while the *bla*_KPC-33_ clone showed 16 mg/L. This potential stepwise trajectory was evident for *bla*_KPC-2_, *bla*_KPC-33_, and *bla*_KPC-145_ as a linear evolutionary path, whereas *bla*_KPC-71_ harbored an independent insertion mutation (c.547_548insATC, resulting in p.S182_P183insS) outside this pathway. Molecular docking analysis predicted variant-specific differences in avibactam binding to the four KPC enzymes (Fig. 3C). Avibactam adopted distinct binding orientations and interacted with different surrounding residues in each variant, compared to KPC-2. This highlights the structural impact that these amino acid changes have on the binding of the inhibitor. Considering that the patient was treated with low-dose ceftazidime-avibactam, we, therefore, performed time-kill assays to evaluate its bactericidal activity against the *bla*_KPC-2_-harboring *K. pneumoniae* (Fig. 3D). Ceftazidime-avibactam exhibited clear concentration-dependent activity, in which 4×MIC and 2×MIC achieved bactericidal effects, 1×MIC caused initial killing followed by regrowth, while sub-MIC levels only inhibited growth without bactericidal activity.

### Coexistence of *K. pneumoniae* strains harboring distinct *bla*_KPC_ variants

**Fig 4:**
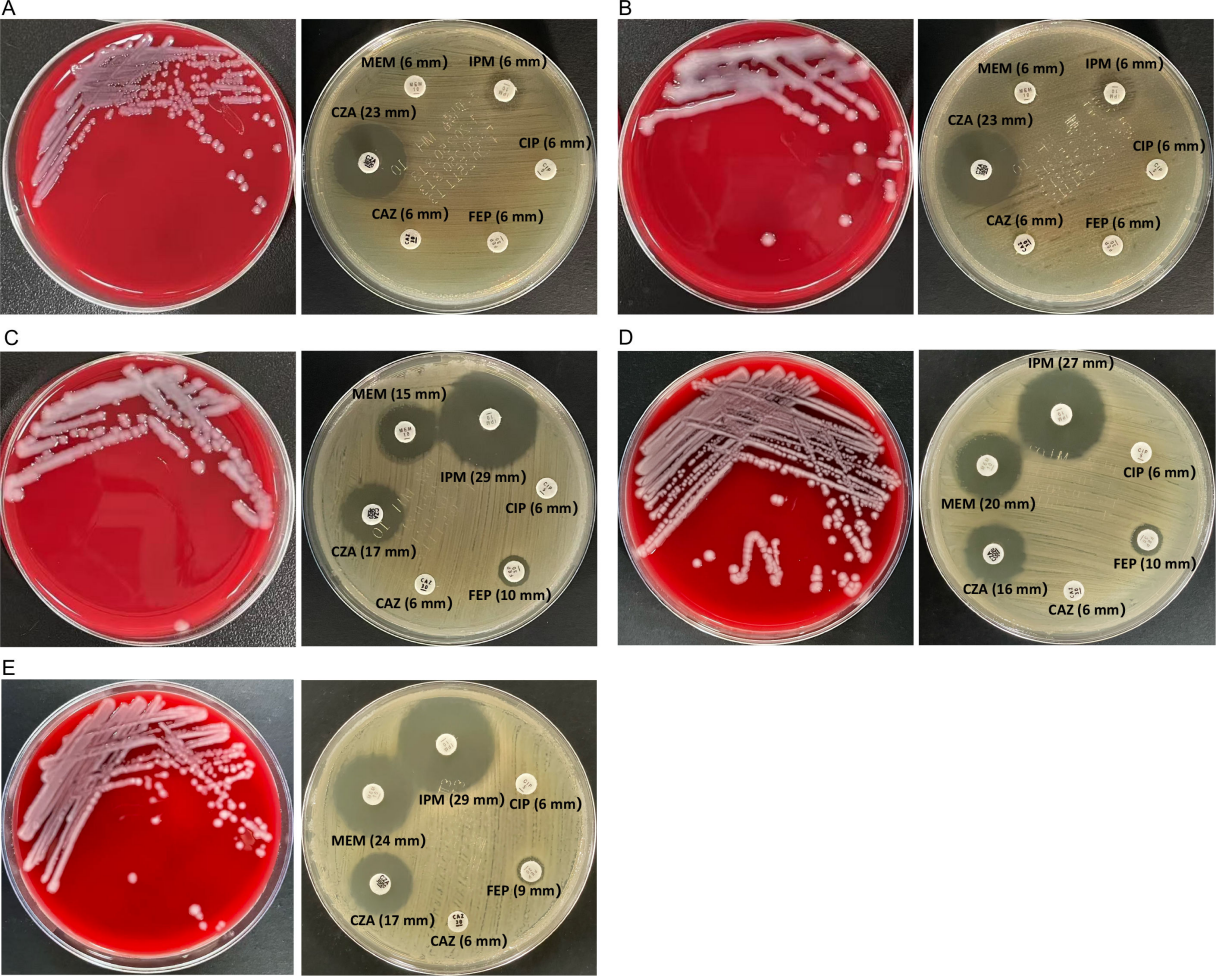
Three *K. pneumoniae* colonies with different morphology, antimicrobial susceptibility profiles, and *bla*_KPC_ subtypes recovered from an archived sputum specimen, beyond the 10 isolates analyzed in this study. From a preserved sputum specimen of the same patient, three morphologically distinct colonies were identified, including a predominant small non-mucoid colony (**A**), a mucoid colony with a positive string test (**B**), and another mucoid colony with a negative string test (**C**). The left panels show colony morphology on blood agar, and the right panels display the corresponding disk diffusion susceptibility profiles. Molecular analysis revealed that isolates A and B carried *bla*_KPC-2_, whereas isolate C carried *bla*_KPC-145_. For completeness, *bla*_KPC-33_ from KPN2 (**D**) and *bla*_KPC-71_ from KPN4 (**E**), the other two subtypes identified in this study, are also shown.

Retrospective review of laboratory reports revealed that two distinct resistance phenotypes of *K. pneumoniae* isolates (KPN19 and KPN20) had been reported on hospital day 85 (19 August). Subsequent genotypic analysis confirmed that they carried *bla*_KPC-2_ and *bla*_KPC-145_, respectively (Fig. 1). This finding raised the possibility that multiple *bla*_KPC_ subtypes might have coexisted within the host during hospitalization. To investigate this, we searched for archived sputum specimens from the same patient and identified 3 specimens that had been cryopreserved for other purposes in addition to the 10 sequential isolates analyzed in this study. Of these, two were negative by culture and PCR, likely due to inappropriate storage conditions. In contrast, the revival of the third specimen (collected on 21 July 2024) yielded three colonies with distinct morphologies. The predominant colony was small and non-mucoid (Fig. 4A), while the other two were mucoid, with one showing a positive string test (Fig. 4B) and the other a negative string test (Fig. 4C). Disk diffusion susceptibility showed divergent resistance profiles, with two isolates being imipenem-resistant/ceftazidime-avibactam-susceptible (Fig. 4A and B) and one imipenem-susceptible/ceftazidime-avibactam-resistant (Fig. 4C). Molecular analysis confirmed that they carried *bla*_KPC-2_ and *bla*_KPC-145_, respectively. For these three revived colonies, only colony morphology and the string test were evaluated, while capsule type was not determined. The minority morphotypes had escaped detection in routine laboratory procedures, likely because the predominant small non-mucoid colonies overgrew and masked them.

## DISCUSSION

Infections caused by KPC-producing *K. pneumoniae* have become a major global health threat due to the rising prevalence and limited treatment options (14, 15). Newly developed β-lactam/β-lactamase inhibitor combinations are preferred agents, with ceftazidime-avibactam being the most widely used in clinical practice (5). However, the emergence of *bla*_KPC_ variants poses a new challenge (3, 6). *bla*_KPC_ mutations acquired during therapy can alter susceptibility phenotype, compromise the activity of β-lactamase inhibitors, and ultimately lead to treatment failure (3, 13). These challenges underscore the urgent need to elucidate the evolutionary dynamics of *bla*_KPC_ and to develop effective therapeutic strategies against *bla*_KPC_ variant-mediated infections. In this study, we characterized the within-host evolution of *K. pneumoniae* harboring *bla*_KPC_ during prolonged antimicrobial treatment. The findings highlight two key factors underlying the persistence of infection: (i) the recurrent and reversible shifts between *bla*_KPC-2_ and its three variants under alternating reduced-dose ceftazidime-avibactam and carbapenem exposure and (ii) the coexistence of *K. pneumoniae* isolates harboring distinct *bla*_KPC_ subtypes within the host.

The evolution from *bla*_KPC-2_ to its variant represents an important adaptive strategy of *K. pneumoniae* to survive antibiotic pressure (11, 13). In this study, three *bla*_KPC-2_ variants (*bla*_KPC-33_, *bla*_KPC-71_, and *bla*_KPC-145_) emerged sequentially during ceftazidime-avibactam exposure. These variants may have arisen through stepwise accumulation of mutations, with *bla*_KPC-33_ carrying a substitution in the Ω loop region (D179Y), while *bla*_KPC-145_ acquired an additional substitution (T264A) based on *bla*_KPC-33_. Molecular docking predicted variant-specific avibactam binding profiles consistent with previous study, which showed that mutations in the *bla*_KPC_ gene can alter the shape of the avibactam-binding pocket (16). Functional assays confirmed that KPC-145 conferred higher ceftazidime-avibactam MICs than KPC-33, indicating that cumulative mutations further enhance resistance. Similar cumulative mutations have been reported in other variants, such as stepwise evolution from *bla*_KPC-2_ to *bla*_KPC-79_ (262V_268N dup) and further to *bla*_KPC-76_ (262V_268N dup plus D179Y), which also led to increased ceftazidime-avibactam MICs (17). A key feature in the current study was that all ceftazidime-avibactam and carbapenem regimens were administered at reduced doses due to renal impairment. We hypothesize that this suboptimal exposure was a major factor driving the recurrent emergence of *bla*_KPC_ variants. Supporting this, the unusually frequent appearance of three variants in a single patient contrasts with the one or two variants typically reported in previous studies (12, 13). While most prior clinical reports did not provide dosing details, an *in vitro* study has demonstrated that inadequate concentrations of ceftazidime-avibactam are more likely to select for *bla*_KPC-2_ mutations (18). Consistent with this, our time-kill assays confirmed that suboptimal concentrations of ceftazidime-avibactam failed to achieve sustained bactericidal activity, resulting in regrowth, which provides a plausible explanation for the *in vivo* selection of resistant variants. Furthermore, reduced-dose imipenem may have created additional sublethal selective pressure that suppressed but failed to eradicate *bla*_KPC_ variants, thereby facilitating their persistence (19), as evidenced by the coexistence of multiple *bla*_KPC_ variants within the patient. Taken together, our findings raise the possibility that reduced-dose ceftazidime-avibactam and imipenem exposure can accelerate *bla*_KPC_ evolution *in vivo*, highlighting the need for validation in additional clinical cases and for optimized dosing strategies to prevent resistance development. In addition, 2 of the 10 isolates studied exhibited a mucoid phenotype despite showing no clear genotypic differences from the other *K. pneumoniae* isolates. This suggests that the hypermucoviscosity may be driven by environmental or regulatory factors rather than genetic changes. However, the underlying mechanism requires further investigation.

The coexistence of *K. pneumoniae* isolates carrying distinct *bla*_KPC_ variants further complicated the treatment, suggesting potential heteroresistance within the host that was not recognized during therapy. Heteroresistance is defined as a population-wide variation in antibiotic resistance, whereby subpopulations within an isolate exhibit different levels of resistance to a given antimicrobial agent. The gold standard for confirming heteroresistance is population analysis profiling (PAP) (20). Reported mechanisms of β-lactam heteroresistance in *K. pneumoniae* mainly involve porin alterations and β-lactamase gene hyperexpression or copy number flexibility (21–23), while heteroresistance mediated by *bla*_KPC_ variants affecting both carbapenem and ceftazidime-avibactam has rarely been described. Recently, Gabriele et al. reported the coexistence of KPC-33- and KPC-14-producing *K. pneumoniae* after prolonged exposure to ceftazidime-avibactam, which was identified in isolates recovered from two paired blood culture bottles (24). Such coexisting subpopulations are otherwise difficult to recognize in routine microbiological workup, as dominant colonies may obscure minority ones. Our findings highlight that close inspection of colony morphology may provide important clues, particularly in cases of persistent infection or atypical resistance phenotypes. The alternating imipenem-susceptible and ceftazidime–avibactam-resistant phenotypes observed in this case may suggest heteroresistance. However, this cannot be confirmed because the PAP was not performed.

Our findings may also provide useful insights for managing infections caused by KPC- or KPC variant-producing *K. pneumoniae*, particularly in cases with reversible resistance phenotypes. In this patient, reversible *bla*_KPC_ mutations were no longer detected after 8 days of combined imipenem and ceftazidime-avibactam therapy, suggesting the potential benefit of this combination. According to the IDSA guidance for antimicrobial-resistant Gram-negative infections, meropenem-vaborbactam, ceftazidime-avibactam, and imipenem-relebactam are the preferred treatment options for infections caused by KPC-producing Enterobacterales (5). In our study, isolates carrying the KPC variant were resistant to ceftazidime-avibactam. However, *in vitro* susceptibility testing demonstrated that meropenem-vaborbactam, imipenem-relebactam, and aztreonam-avibactam retained activity against both *bla*_KPC-2_ and its variants. These findings support their potential role as alternative therapeutic options where available. Taken together, our results highlight the need for further clinical data and standardized treatment strategies for managing infections caused by KPC/KPC variant-producing *K. pneumoniae*.

This study has several limitations. First, the precise mechanisms underlying the high mutational frequency of *bla*_KPC_ in this case remain unclear. Although antibiotic pressure was likely a key driver, host-related factors or bacterial characteristics may also have contributed. Second, functional validation of *bla*_KPC_ variants, including their impact on bacterial fitness and virulence, was not performed. In addition, although it is known that disruptions in OmpK35/OmpK36 influence β-lactam susceptibility, we did not conduct in-depth analyses of porin alterations or their functional consequences. Further investigation of these aspects is required. Third, as the PAP test was not performed, it was not possible to definitively distinguish true heteroresistance from the sequential emergence of coexisting subpopulations or potential reinfection events. In conclusion, this study demonstrates that the observed stepwise cumulative *bla*_KPC_ mutations and the coexistence of *K. pneumoniae* isolates harboring distinct *bla*_KPC_ subtypes contributed to persistent infection and that reduced-dose ceftazidime-avibactam and carbapenem likely facilitated *bla*_KPC_ evolution. The findings underscore the difficulty in managing infections caused by KPC/KPC variant-producing *K. pneumoniae* and highlight the need for more clinical data to support the development of evidence-based treatment guidance. Newer β-lactam/β-lactamase inhibitor combinations (meropenem-vaborbactam, imipenem-relebactam, and aztreonam-avibactam) and the combination of imipenem and ceftazidime-avibactam may represent promising therapeutic options although further clinical validation is required.

## MATERIALS AND METHODS

### Clinical course

The patient was admitted on 26 May 2024 for management of a chronic obstructive pulmonary disease exacerbation and showed no evidence of active infection on admission. On hospital day 7, the patient developed a fever and a productive cough, and empirical meropenem therapy (500 mg, intravenous infusion, every 8 hours) was initiated (Fig. 1). On day 8, sputum culture yielded *K. pneumoniae* susceptible to imipenem but resistant to ceftazidime-avibactam. Four days later, an imipenem-resistant but ceftazidime-avibactam-susceptible *K. pneumoniae* strain was cultured, and no apparent clinical improvement was observed. Antimicrobial treatment was switched to ceftazidime-avibactam (0.94 g, intravenous infusion, every 12 hours), with dosing adjustment for renal impairment (eGFR fluctuated between 29 and 42 mL/min/1.73 m^2^ during hospitalization). During the subsequent 4-month hospitalization, imipenem-susceptible, ceftazidime-avibactam-resistant *K. pneumoniae* strains were alternately isolated with imipenem-resistant, ceftazidime-avibactam-susceptible strains over distinct periods (Fig. 1). The microbiological shifts were accompanied by recurrent low-grade fever, productive cough, and elevated inflammatory markers. Antimicrobial therapy was guided by susceptibility profiles and clinical signs. The patient received reduced-dose ceftazidime-avibactam (1.25 g, intravenous infusion, every 8 h) and imipenem (500 mg, intravenous infusion, every 8 h initially, later adjusted to every 6 h) during different treatment periods, with partial overlap in use. Tigecycline (50 mg, intravenous infusion, every 12 h) was intermittently used in combination with either agent. Oxygen saturation remained consistently above 95% throughout the entire hospitalization. Persistent infection was defined as repeated isolation of the same species as the initial strain from clinical specimens, accompanied by ongoing symptoms, for at least 2 months. A total of 10 non-duplicate *K. pneumoniae* isolates were collected for further analysis, each representing a distinct phenotypic shift observed during the course of infection (Fig. 1). Notably, the isolate dates indicate the collection time of clinical specimens, not identification report dates. The patient was eventually discharged on hospital day 122 after clinical improvement and negative sputum cultures.

### Bacterial phenotypic analysis

Bacterial species identification was performed using matrix-assisted laser desorption/ionization-time of flight mass spectrometry (MALDI-TOF MS; bioMérieux, Marcy-l’Étoile, France). Antimicrobial susceptibility testing was performed by the broth microdilution method according to the Clinical and Laboratory Standards Institute (CLSI) M07 guidelines (25). Minimum inhibitory concentrations (MICs) were interpreted using the 2025 CLSI breakpoints (26). For agents without CLSI criteria, aztreonam-avibactam was interpreted using the EUCAST breakpoint (susceptible: ≤4/4 mg/L; resistant: ≥8/4 mg/L), eravacycline using the ChiCAST susceptible-only breakpoint (susceptible: ≤1 mg/L), and tigecycline using the U.S. Food and Drug (FDA) criteria (susceptible: ≤2 mg/L; resistant: ≥8 mg/L). Carbapenemase production was screened using a colloidal gold immunoassay (Dynamiker Biotechnology, Tianjin, China).

Efflux activity was evaluated by performing MIC testing in the presence of 16 mg/L carbonyl cyanide m-chlorophenylhydrazone (CCCP) or 20 mg/L phenylalanine-arginine β-naphthylamide (PAβN), and a ≥4-fold MIC decrease was considered a positive efflux phenotype. *Escherichia coli* ATCC 25922 and *K. pneumoniae* NCTC 1705 (*bla*_KPC_ positive) were used as quality control. In addition, during the retrospective analysis of this case, we found that two *bla*_KPC_ subtypes were isolated from a sputum specimen collected on hospital day 85 (Fig. 1). To investigate the potential coexistence of *K. pneumoniae* strains harboring distinct *bla*_KPC_ subtypes, several preserved sputum specimens were retrieved and revived. Time-kill assays were performed in cation-adjusted Mueller–Hinton broth using an initial inoculum of 5 × 10^5^ CFU/mL. Ceftazidime-avibactam was tested at five concentrations relative to the MIC (1/4×, 1/2×, 1×, 2×, and 4× MIC). Samples were taken at 0, 2, 4, 6, 8, 10, and 24 h, serially diluted, and plated for colony counts.

### Whole-genome sequencing and bioinformatics analysis

Ten *K. pneumoniae* isolates, each representing a shift in resistance phenotype, were subjected to whole-genome sequencing. Genomic DNA was extracted using the QIAamp DNA Mini Kit (Qiagen, Hilden, Germany) following the manufacturer’s instructions. Whole-genome sequencing was performed on the Illumina NovaSeq XPlus platform (Illumina Inc., CA, USA). Raw reads were assembled into draft genomes using SOAPdenovo2 (version 2.04) (27). Genome annotation was performed using Prokka (version 1.14.6) (28). Antimicrobial resistance genes were identified using ResFinder (version 4.7.2) (29). *bla*_KPC_ subtypes were confirmed by BLAST analysis against the National Center for Biotechnology Information (NCBI) reference gene database. Multilocus sequence typing (MLST) and capsular (K) type were determined using Kleborate (version 3.1.3) (30). Plasmid replicon types were identified using PlasmidFinder (version 2.0.1) based on ≥95% identity and ≥60% coverage following the default criteria (31). Core-genome alignment was generated using Roary (version 3.13.0) to provide an overview of core-genome single-nucleotide polymorphism (SNP) diversity among isolates (32). For the phylogenetic analysis, SNPs were identified using Snippy (version 4.6.0, https://github.com/tseemann/snippy) with *K. pneumoniae* KPN2 as the reference genome. A reference-guided core genome alignment was then generated using Snippy-core. This alignment was analyzed using Gubbins (version 3.4.3) to obtain a recombination-free data set (33), and the resulting alignment was used to infer a maximum likelihood phylogeny in RAxML-NG (version 1.2.2) (34). Phylogenetic visualization was performed using Chiplot (https://www.chiplot.online). For the molecular docking analysis, KPC-2 and its three variants were modeled using AlphaFold3 (35), and the avibactam structure was obtained from PubChem (https://pubchem.ncbi.nlm.nih.gov). Docking was performed using CB-Dock (36). The top-ranked pose for each variant was selected for comparison. The docked complexes were then aligned and analyzed in PyMOL to examine differences in active-site interactions

### Plasmid conjugation, transformation, and *bla*_KPC_ cloning assays

To investigate the characteristics of the different *bla*_KPC_-harboring plasmids, plasmid conjugation, transformation, and cloning assays were performed. For the conjugation assay, *E. coli* EC600 or J53 were used as recipient strains. No *bla*_KPC_-positive transconjugants were obtained, indicating the *bla*_KPC_-harboring plasmids in this study are not self-conjugative. For the transformation assay, plasmid DNA was extracted from the donor strain and introduced into *E. coli* DH5α by electroporation (MicroPulser; Bio-Rad, CA, USA). Transformants were selected on Mueller-Hinton agar plates supplemented with ampicillin (50 mg/L), and the presence of *bla*_KPC_ was confirmed by PCR and Sanger sequencing. For the cloning assay, the *bla*_KPC_ genes, including their putative promoter, were amplified using specific primer pairs: EcoR1-KPC-F (5′-CCATGATTACGAATTCGAGACAATAACCCTGATAAATGCTTCAATAATATTGAAAAAATG-3′) and BamH1-KPC-R (5′-CGACTCTAGAGGATCCTTACTGCCCGTTGACGCCC-3′). The PCR products were purified and cloned into the pHSG398 vector (Sangon Biotech, Shanghai, China). The recombinant plasmids (pKPC-2, pKPC-33, pKPC-71, and pKPC-145) were introduced into *E. coli* DH5α by chemical transformation. MICs of the transconjugants and recombinant clones were determined by the broth microdilution method.

## Data Availability

The genome data of the 10 clinical *K. pneumoniae* isolates have been deposited in the NCBI database under BioProject accession number PRJNA1312957.

## SUPPLEMENTAL MATERIAL

Fig. S1 spectrum.03308-25-s0001.pdf Colony morphology of the 10 clinical *K. pneumoniae* isolates.

Table S1 spectrum.03308-25-s0002.xlsx Efflux pump inhibition assays showed negative results.

ASM does not own the copyrights to Supplemental Material that may be linked to, or accessed through, an article. The authors have granted ASM a non-exclusive, world-wide license to publish the Supplemental Material files. Please contact the corresponding author directly for reuse.
