# Supplementary material for: Suboptimal ceftazidime-avibactam exposure drives sequential blaKPC mutations and intra-host coexistence of Klebsiella pneumoniae harboring distinct variants leading to persistent infection
Source: Microbiol Spectr. 2026 Feb 20;14(4):e03308-25. doi: 10.1128/spectrum.03308-25 (PMC13055305; doi:10.1128/spectrum.03308-25)
Supplement: Fig. S1 — Colony morphology of the 10 clinical K. pneumoniae isolates. [file spectrum.03308-25-s0001.pdf]

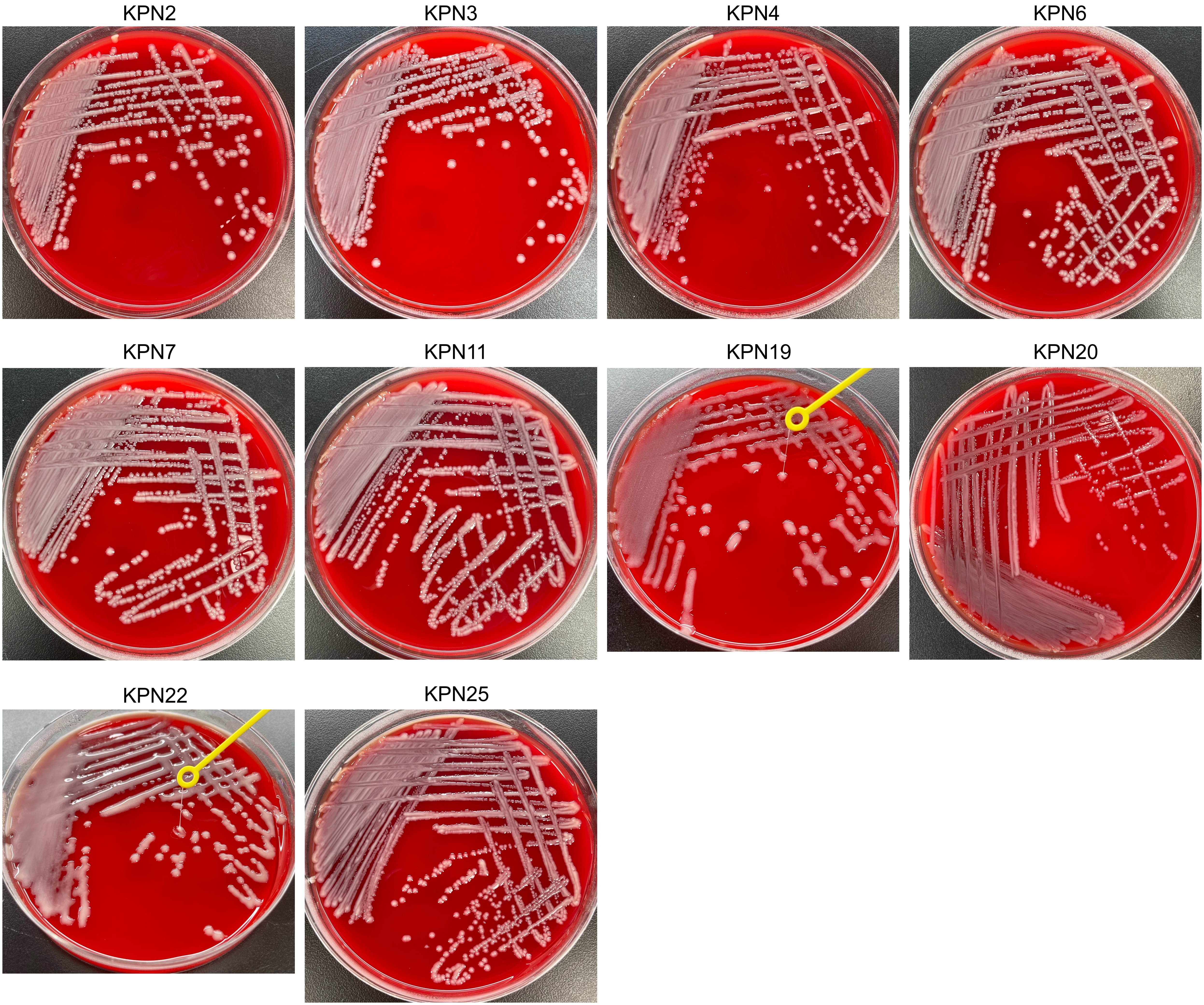

Supplemental Figure 1. Colony morphology of the ten clinical *K. pneumoniae* isolates

KPN19 and KPN22 (both carrying *bla*$_{\mathrm{KPC-2}}$) showed a mucoid phenotype and were positive in the string test, and the other isolates were non-mucoid.
